# Capturing vitality: Toward a multidimensional lens for intrinsic capacity and healthy aging

**DOI:** 10.1016/j.jnha.2025.100636

**Published:** 2025-07-23

**Authors:** Hussein Samhat, Gustavo Duque

**Affiliations:** a Simone & Edouard Schouela RUISSS McGill Centre of Excellence for Sustainable Health of Seniors (Schouela CEDurable), Montréal, QC, Canada; b Bone, Muscle & Geroscience Group, Research Institute of the McGill University Health Centre, Montréal, Québec, Canada

The global population is aging at an unprecedented pace [1]. By 2030, one in six people worldwide will be aged 60 or older [2]. Between 2020 and 2050, the number of people aged 60 and older is expected to double to 2.1 billion, while those aged 80 and over are projected to triple, reaching 426 million [2]. In response, the United Nations declared 2021–2030 the "Decade for Healthy Aging", with the World Health Organization (WHO) leading global efforts to improve the lives of older people, their families, and their communities [3].

The WHO calls for aging science to move beyond disease-centric models into more holistic, person-centered approaches. This aligns with its definition of healthy aging as “the process of developing and maintaining the functional ability that enables wellbeing in older age” [1]. To this end, the WHO proposed using functional ability and intrinsic capacity (IC) as key outcome indicators (Fig. 1). Functional ability thus refers to all the health-related attributes that allow people to meet their goals and live meaningfully, resulting from the dynamic interaction between a person’s IC and their environment [1].

**Fig. 1:**
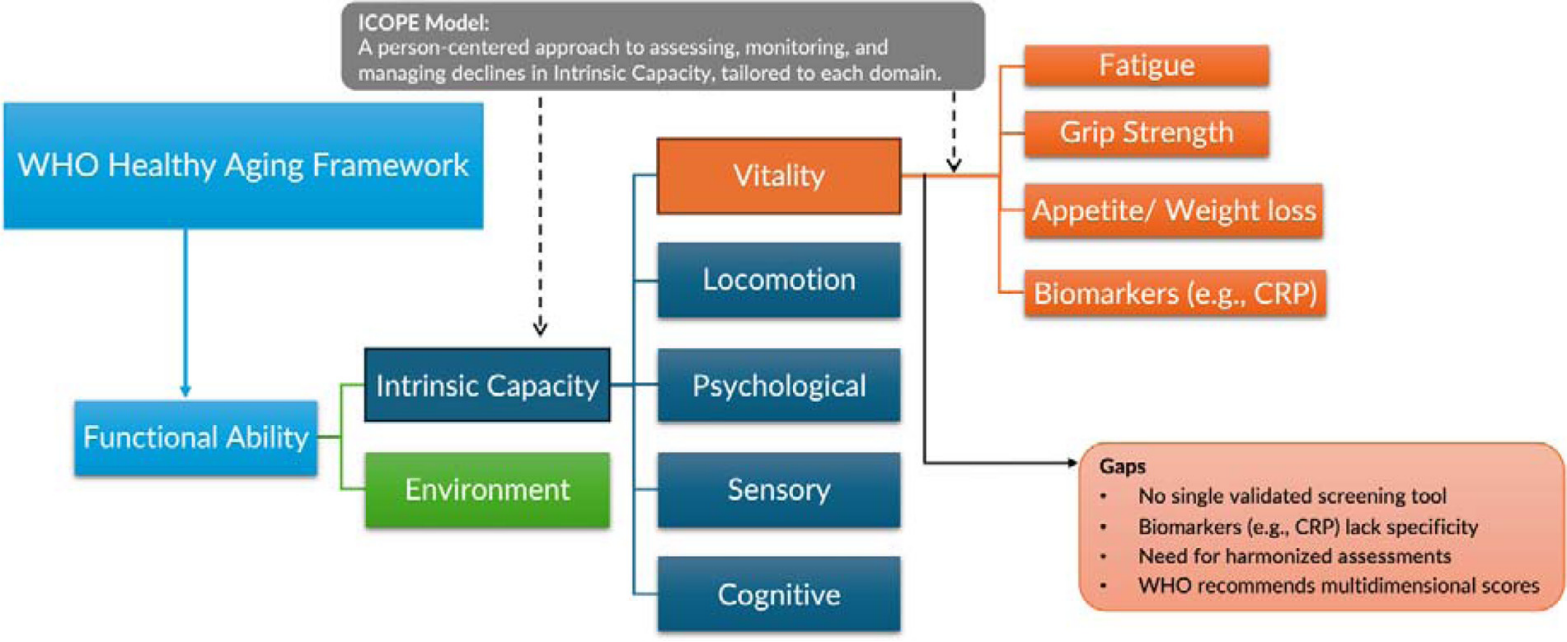
Positioning Vitality within the Framework for Healthy Aging and Intrinsic Capacity. Diagram showing vitality's place within the broader WHO Healthy Aging framework, illustrating the relationship between Functional Ability, Intrinsic Capacity (IC), and the Integrated Care for Older People (ICOPE) model. Vitality, a key IC domain, is described by important indicators and current gaps.

IC encompasses all the physical and mental capacities that a person can draw on at any point in time. It consists of five domains: vitality, locomotion, psychological, sensory, and cognitive [1] (Fig. 1). While IC is the conceptual framework of *why* and *what* to measure, the WHO’s Integrated Care for Older People (ICOPE) model provides the *how*; to assess, monitor, and manage declines in IC through structured, person-centered interventions tailored to each domain [4]. Among the five domains, vitality remains the least clearly defined. Often used as a proxy for metabolic or physiological reserve, it lacks standardized operational tools (Fig. 1).

In the 2024 ICOPE report, vitality is reaffirmed as a critical domain of IC, typically assessed through indicators such as unintentional weight loss, fatigue, poor appetite, mobility, and grip strength [4,5]. However, no single tool is validated for routine screening, particularly in primary care. Biomarkers like C-reactive protein (CRP) have been explored in research but are not yet suitable for clinical use due to their limited specificity [6]. The WHO Vitality Capacity Working Group (2019) has emphasized the need to clearly define vitality, harmonize screening methods across settings, and validate multidimensional assessment tools that can reliably capture its complexity [7].

In this context, the study by Peng et al. [8] offers a timely and nuanced investigation of vitality. Using data from the Gan-Dau Healthy Longevity Plan in Taiwan, the authors assess how four vitality-related attributes (nutritional status, fatigue, grip strength, and inflammation) relate to other IC domains and to psychosocial constructs such as resilience and happiness, both increasingly recognized as key dimensions of aging well [8]. Notably, self-perceived fatigue emerged as the most powerful marker of decline across multiple IC domains. It was associated with greater odds of locomotion impairment (adjusted OR = 2.47, [95% CI 1.4–4.2]) and psychological impairment (aOR = 8.34 [95% CI 4.8–14.4]), as well as markedly lower scores on resilience and happiness scales [8]. These findings align with recent calls to treat vitality not as a fixed trait but as a dynamic profile of physical and emotional energy [9].

Their study underscores the variability in vitality expression, showing that characteristics like low grip strength (38.2% prevalence) and poor nutritional status (15.4%) are common, while others such as self-perceived fatigue (6.7%) and high CRP levels (6.4%) are less frequent but may be more helpful in distinguishing and predicting adverse outcomes. However, these tests' application in clinical settings could be hindered by their complexity and cost. Consequently, real-world screening studies, like the INSPIRE ICOPE-Care program, found that appetite and weight loss—used as markers for vitality—were strongly linked to impairments across all IC domains, especially locomotion and mental health [9]. Monitoring these measures (with or without grip strength) in clinical practice could help assess vitality and prompt therapeutic actions.

## 1 Expanding the scope: resilience and happiness

One of the most significant contributions of this study is its inclusion of positive psychosocial constructs, specifically resilience and happiness. These dimensions are not currently part of the ICOPE framework, although happiness could be incorporated into the mood component of IC. Peng et al. demonstrate that vitality impairments, particularly fatigue and nutritional deficits, are strongly linked to reduced psychosocial well-being [8]. By connecting vitality not only to physical function but also to psychological flourishing, the authors help redefine vitality as a two-way link between body and mind. This has practical implications: resilience and happiness may not only be the results of decreased vitality but could also influence its course.

The inclusion of these domains aligns with emerging geroscience literature that considers resilience, defined as the ability to resist or recover from physiological stress, as a fundamental factor influencing aging trajectories [10]. Although it is challenging to measure, resilience is now recognized as an important mediator between biological aging and clinical function. Overall, these findings imply that a composite score, especially one that combines fatigue and muscle strength, could provide a practical approach to assessing vitality in real-world settings.

Several studies have already linked vitality impairments to increased mortality, medication burden [11], and higher frailty risk [12]. Evidence also suggests that strengthening IC domains through behavioral or pharmacologic interventions may reduce functional decline [12]. The IC-resilience axis may ultimately serve as a clinical endpoint in geroscience trials aiming to extend healthspan [10].

## 2 Toward practical measurement

From a clinical perspective, the study by Peng et al. [8] highlights the potential value of simple, feasible tools for vitality screening. Grip strength measurement is inexpensive, portable, and widely validated across aging populations. The Mini Nutritional Assessment-Short Form (MNA-SF) provides a reliable nutritional screen [13], especially in community settings. Fatigue, although subjective, may indicate early physiological decline and therefore warrants routine assessment.

However, operationalization matters. As Lu et al. recently demonstrated, the choice of vitality assessment method (e.g., grip strength vs. fatigue vs. nutritional intake) yields significant variability in impairment detection and population stratification [14]. Similarly, in a large cohort study in France, Gaussens et al. found that appetite and weight loss were independently associated with deficits across cognition, locomotion, sensory, and psychological domains, supporting their use in scalable vitality screening [9].

## 3 Implications for research, practice, and policy

Peng et al.’s study contributes meaningfully to clarifying how vitality can, and should, be measured. It also raises questions for the field: A) Should fatigue be treated as an early warning sign of IC decline, warranting early intervention? B) Is grip strength the most scalable “entry point” into vitality screening for primary care or community health programs? C) How might resilience and happiness be better integrated into functional aging models, not as outcomes alone but as potential inputs?

As vitality remains a core yet underdeveloped domain within ICOPE, these findings reinforce the need for more longitudinal studies, cross-cultural validation, and intervention trials that treat vitality as a modifiable capacity, responsive to physical activity, nutrition, and psychosocial support.

## 4 Conclusion

The study by Peng et al. [8] brings us closer to defining vitality as a practical, multidimensional aspect central to healthy aging. Instead of a fixed measure of biological reserves, vitality is seen here as a fluid profile that includes strength, nutrition, fatigue, and affect. As aging science moves beyond disease-focused models, adopting holistic, person-centered approaches like this will be essential. Vitality is not a single figure; it reflects function, context, and well-being. Peng et al. provide a clearer way to understand that concept and, more importantly, to act on it.

## Declaration of competing interest

The author declares no conflicts of interest.
